# Supplementary material for: Catecholaminergic neuromodulation and selective attention jointly shape perceptual decision-making
Source: eLife. 2023 Dec 1;12:RP87022. doi: 10.7554/eLife.87022 (PMC10691802; doi:10.7554/eLife.87022)
Supplement: MDAR checklist [file elife-87022-mdarchecklist1.docx]

**Materials Design Analysis Reporting (MDAR)**

**Checklist for Authors**

The [MDAR framework](https://osf.io/xfpn4/) establishes a minimum set of requirements in transparent reporting mainly applicable to studies in the life sciences.

*eLife* asks authors to **provide detailed information within their article** to facilitate the interpretation and replication of their work. Authors can also upload supporting materials to comply with relevant reporting guidelines for health-related research (see [EQUATOR Network](http://www.equator-network.org/%20)), life science research (see the [BioSharing Information Resource](http://biosharing.org/)), or animal research (see the [ARRIVE Guidelines](http://www.plosbiology.org/article/info:doi/10.1371/journal.pbio.1000412) and the [STRANGE Framework](https://doi.org/10.1038/d41586-020-01751-5); for details, see *eLife*’s [Journal Policies](https://reviewer.elifesciences.org/author-guide/journal-policies)). Where applicable, authors should refer to any relevant reporting standards materials in this form.

For all that apply, please note **where in the article** the information is provided. Please note that we also collect information about data availability and ethics in the submission form.

**Materials:**

| **Newly created materials** | **Indicate where provided: section/figure legend** | **N/A** |
| --- | --- | --- |
| The manuscript includes a dedicated "materials availability statement" providing transparent disclosure about availability of newly created materials including details on how materials can be accessed and describing any restrictions on access. |  | N/A |
|  |  |  |
| **Antibodies** | **Indicate where provided: section/figure legend** | **N/A** |
| For commercial reagents, provide supplier name, catalogue number and [RRID](https://scicrunch.org/resources), if available. |  | N/A |
|  |  |  |
| **DNA and RNA sequences** | **Indicate where provided: section/figure legend** | **N/A** |
| Short novel DNA or RNA including primers, probes: Sequences should be included or deposited in a public repository. |  | N/A |
|  |  |  |
| **Cell materials** | **Indicate where provided: section/figure legend** | **N/A** |
| Cell lines: Provide species information, strain. Provide accession number in repository OR supplier name, catalog number, clone number, OR RRID. |  | N/A |
| Primary cultures: Provide species, strain, sex of origin, genetic modification status. |  | N/A |
|  |  |  |
| **Experimental animals** | **Indicate where provided: section/figure legend** | **N/A** |
| Laboratory animals or Model organisms: Provide species, strain, sex, age, genetic modification status. Provide accession number in repository OR supplier name, catalog number, clone number, OR RRID. |  | N/A |
| Animal observed in or captured from the field: Provide species, sex, and age where possible. |  | N/A |
|  |  |  |
| **Plants and microbes** | **Indicate where provided: section/figure legend** | **N/A** |
| Plants: provide species and strain, ecotype and cultivar where relevant, unique accession number if available, and source (including location for collected wild specimens). |  | N/A |
| Microbes: provide species and strain, unique accession number if available, and source. |  | N/A |
|  |  |  |
| **Human research participants** | **Indicate where provided: section/figure legend) or state if these demographics were not collected** | **N/A** |
| If collected and within the bounds of privacy constraints report on age, sex, gender and ethnicity for all study participants. | All participants were aged between 18 and 30. The sex of all participants was male. We did not collect data about gender and ethnicity. |  |

**Design:**

| **Study protocol** | **Indicate where provided: section/figure legend** | **N/A** |
| --- | --- | --- |
| If the study protocol has been pre-registered, provide DOI. For clinical trials, provide the trial registration number OR cite DOI. |  | N/A |
|  |  |  |
| **Laboratory protocol** | **Indicate where provided: section/figure legend** | **N/A** |
| Provide DOI OR other citation details if detailed step-by-step protocols are available. |  | N/A |
|  |  |  |
| **Experimental study design (statistics details) *** | | |
| **For in vivo studies: State whether and how the following have been done** | **Indicate where provided: section/figure legend. If it could have been done, but was not, write “not done”** | **N/A** |
| Sample size determination | We did not perform a formal sample size determination. The sample size of this study (N=30) was based on sample sizes in previously reported comparable studies using the same pharmacological agents in a non-clinical setting. We did not report this procedure in our manuscript. |  |
| Randomisation | Drug order was randomized across participants. Randomization was performed by the pharmacy that provided the pharmaceutical agents and was not revealed to the researchers until completion of data collection.  We report that the drug order was randomized in **Materials and Methods**, under **Drug administration**. |  |
| Blinding | Drug administration was double-blind, thus neither participants nor the researchers were aware of the drug order. Blinding was performed by the pharmacy and not revealed to the researchers until completion of data collection.  We report on the double-blind nature of this study in **Materials and Methods**, under **Drug administration**. |  |
| Inclusion/exclusion criteria | We used the following inclusion and exclusion criteria:  Inclusion criteria:  ● Healthy volunteers between 18 and 30 years old;  ● Native Dutch speakers;  ● Predominant right-handedness;  ● BMI between 18.5 and 30.  ● Male  Exclusion criteria:  ● Allergy for atomoxetine, donepezil, memantine, or one of the inactive ingredients of these products;  ● (History of) psychiatric treatment;  ● First-degree relative with (history of) schizophrenia or major depression;  ● (History of) clinically significant hepatic, cardiac, obstructive respiratory, renal, cerebrovascular, metabolic or pulmonary disease, including, but not limited to fibrotic disorders;  ● Claustrophobia;  ● Regular usage of medicines (antihistamines or occasional use of paracetamol);  ● (History of) neurological disease;  ● (History of) epilepsy;  ● Abnormal hearing or (uncorrected) vision;  ● Average use of more than 15 alcoholic beverages weekly;  ● Smoking  ● History of drug (opiate, LSD, (meth)amphetamine, cocaine, solvents, cannabis, or barbiturate) or alcohol dependence;  ● Any known other serious health problem or mental/physical stress;  ● Used psychotropic medication, or recreational drugs over a period of 72 hours prior to each test session,  ● Used alcohol within the last 24 hours prior to each test session;  ● (History of) pheochromocytoma.  ● Narrow-angle glaucoma;  ● (History of) ulcer disease;  ● Galactose intolerance, Lapp lactase deficiency or glucose­galactose malabsorption.  ● (History of) convulsion;  ● Prolonged QT syndrome;  ● Known degraded metabolism via CY2D6. |  |
|  |  |  |
| **Sample definition and in-laboratory replication** | **Indicate where provided: section/figure legend** | **N/A** |
| State number of times the experiment was replicated in the laboratory. |  | N/A |
| Define whether data describe technical or biological replicates. |  | N/A |
|  |  |  |
| **Ethics** | **Indicate where provided: section/submission form** | **N/A** |
| Studies involving human participants: State details of authority granting ethics approval (IRB or equivalent committee(s), provide reference number for approval. | All procedures were approved by the Medical Ethical Committee (METC) of the Amsterdam University Medical Centre under project number: NL64341.018.18 |  |
| Studies involving experimental animals: State details of authority granting ethics approval (IRB or equivalent committee(s), provide reference number for approval. |  | N/A |
| Studies involving specimen and field samples: State if relevant permits obtained, provide details of authority approving study; if none were required, explain why. |  | N/A |
|  |  |  |
| **Dual Use Research of Concern (DURC)** | **Indicate where provided: section/submission form** | **N/A** |
| If study is subject to dual use research of concern regulations, state the authority granting approval and reference number for the regulatory approval. |  | N/A |

**Analysis:**

| **Attrition** | **Indicate where provided: section/figure legend** | **N/A** |
| --- | --- | --- |
| Describe whether exclusion criteria were pre-established. Report if sample or data points were omitted from analysis. If yes, report if this was due to attrition or intentional exclusion and provide justification. | We excluded two participants because they did not conclude all three drug sessions. From the remaining participants, data from trials in which fixation was lost (eye-blink or gaze position >1.5° visual angle from fixation on the horizontal axis) and in which participants did not respond within the response window (1400ms from stimulus onset) were excluded from further analysis.  We report on these procedures in the **Methods and Materials** under **Participants**, **Data acquisition and preprocessing**, and **Data Analysis**. |  |
|  |  |  |
| **Statistics** | **Indicate where provided: section/figure legend** | **N/A** |
| Describe statistical tests used and justify choice of tests. | For analyses of physiological effects of drug (reported in **Figure 1**, **Figure 1 – Supplement 1**), we performed one-way repeated measures (rm)ANOVAs to establish omnibus drug effects. Post-hoc t-tests to compare pairwise drug effects versus placebo.  For analyses of behavioral data (reported in **Figure 2A-B**, **Figure 2 - Supplement 1**), the CPP analyses (reported in **Figure 3D-F**), cue-locked ERP (reported in **Figure 5B**), and alpha power analyses (reported in **Figure 5E**) we performed 2x3 (cue validity x drug) rmANOVAs to establish main effects of drug condition (omnibus effect), cue validity and their interaction (omnibus effect). Post-hoc 2x2 (cue validity x drug) rmANOVAs were performed to establish pairwise (drug vs. placebo) main drug and interaction effects.  In the case of insignificant p-values, we performed Bayesian analogues of the reported test and provided Bayes Factors indicating evidence for the null-hypothesis (BF_01_).  For statistical analysis of stimulus-locked occipital ERP data, we performed cluster-corrected permutation F-tests (2x2x2 factorial, cue x stimulus identity x drug, 10000 permutations, cluster-threshold p<.05).  The statistical analyses for cue-locked ERP and alpha-power data were similar to above, but excluding the factor stimulus identity as these analyses were performed in the pre-stimulus time-window.  These procedures are reported in the **Materials and Methods**, under **Data analysis**. |  |
|  |  |  |
| **Data availability** | **Indicate where provided: section/submission form** | **N/A** |
| For newly created and reused datasets, the manuscript includes a data availability statement that provides details for access (or notes restrictions on access). | We have included a data availability statement at the beginning of the manuscript, including a DOI where the data will be publicly available after final publication. |  |
| When newly created datasets are publicly available, provide accession number in repository OR DOI and licensing details where available. | See above. |  |
| If reused data is publicly available provide accession number in repository OR DOI, OR URL, OR citation. |  | N/A |
|  |  |  |
| **Code availability** | **Indicate where provided: section/figure legend** | **N/A** |
| For any computer code/software/mathematical algorithms essential for replicating the main findings of the study, whether newly generated or re-used, the manuscript includes a data availability statement that provides details for access or notes restrictions. | Preprocessing and analysis code will be provided alongside the data, under the same DOI. |  |
| Where newly generated code is publicly available, provide accession number in repository, OR DOI OR URL and licensing details where available. State any restrictions on code availability or accessibility. | See above. |  |
| If reused code is publicly available provide accession number in repository OR DOI OR URL, OR citation. |  | N/A |

**Reporting:**

The MDAR framework recommends adoption of discipline-specific guidelines, established and endorsed through community initiatives.

| **Adherence to community standards** | **Indicate where provided: section/figure legend** | **N/A** |
| --- | --- | --- |
| State if relevant guidelines (e.g., ICMJE, MIBBI, ARRIVE, STRANGE) have been followed, and whether a checklist (e.g., CONSORT, PRISMA, ARRIVE) is provided with the manuscript. |  | N/A |

* We provide the following guidance regarding transparent reporting and statistics; we also refer authors to [Ten common statistical mistakes to watch out for when writing or reviewing a manuscript](https://doi.org/10.7554/eLife.48175).

**Sample-size estimation**

- You should state whether an appropriate sample size was computed when the study was being designed
- You should state the statistical method of sample size computation and any required assumptions
- If no explicit power analysis was used, you should describe how you decided what sample (replicate) size (number) to use

**Replicates**

- You should report how often each experiment was performed
- You should include a definition of biological versus technical replication
- The data obtained should be provided and sufficient information should be provided to indicate the number of independent biological and/or technical replicates
- If you encountered any outliers, you should describe how these were handled
- Criteria for exclusion/inclusion of data should be clearly stated
- High-throughput sequence data should be uploaded before submission, with a private link for reviewers provided (these are available from both GEO and ArrayExpress)

**Statistical reporting**

- Statistical analysis methods should be described and justified
- Raw data should be presented in figures whenever informative to do so (typically when N per group is less than 10)
- For each experiment, you should identify the statistical tests used, exact values of N, definitions of center, methods of multiple test correction, and dispersion and precision measures (e.g., mean, median, SD, SEM, confidence intervals; and, for the major substantive results, a measure of effect size (e.g., Pearson's r, Cohen's d)
- Report exact p-values wherever possible alongside the summary statistics and 95% confidence intervals. These should be reported for all key questions and not only when the p-value is less than 0.05.

**Group allocation**

- Indicate how samples were allocated into experimental groups (in the case of clinical studies, please specify allocation to treatment method); if randomization was used, please also state if restricted randomization was applied
- Indicate if masking was used during group allocation, data collection and/or data analysis
